# Uncovering the core principles of the gut-lung axis to enhance innate immunity in the chicken

**DOI:** 10.3389/fimmu.2022.956670

**Published:** 2022-10-04

**Authors:** Vincent Saint-Martin, Pascale Quéré, Sascha Trapp, Rodrigo Guabiraba

**Affiliations:** ISP, INRAE, Université de Tours. 37380, Nouzilly, France

**Keywords:** microbiota, immunity, poultry, chicken, gut-lung axis

## Abstract

Research in mammals has evidenced that proper colonization of the gut by a complex commensal microbial community, the gut microbiota (GM), is critical for animal health and wellbeing. It greatly contributes to the control of infectious processes through competition in the microbial environment while supporting proper immune system development and modulating defence mechanisms at distant organ sites such as the lung: a concept named ‘gut-lung axis’. While recent studies point to a role of the GM in boosting immunity and pathogen resilience also in poultry, the mechanisms underlying this role are largely unknown. In spite of this knowledge gap, GM modulation approaches are today considered as one of the most promising strategies to improve animal health and welfare in commercial poultry production, while coping with the societal demand for responsible, sustainable and profitable farming systems. The majority of pathogens causing economically important infectious diseases in poultry are targeting the respiratory and/or gastrointestinal tract. Therefore, a better understanding of the role of the GM in the development and function of the mucosal immune system is crucial for implementing measures to promote animal robustness in commercial poultry production. The importance of early gut colonization in the chicken has been overlooked or neglected in industrial poultry production systems, where chicks are hampered from acquiring a complex GM from the hen. Here we discuss the concept of strengthening mucosal immunity in the chicken through GM modulation approaches favouring immune system development and functioning along the gut-lung axis, which could be put into practice through improved farming systems, early-life GM transfer, feeding strategies and pre-/probiotics. We also provide original data from experiments with germ-free and conventional chickens demonstrating that the gut-lung axis appears to be functional in chickens. These key principles of mucosal immunity are likely to be relevant for a variety of avian diseases and are thus of far-reaching importance for the poultry sector worldwide.

## Introduction

### State of the art: The holobiont and the host immune system

The concept of the mammalian holobiont - the co-evolutionary assemblage of eukaryotic and prokaryotic elements of an organism - has emerged from the current revolution in our understanding of host–microbial interactions (1). It is today well established that the gut microbiota (GM) - the ensemble of microorganisms inhabiting the gastrointestinal tract - plays a key role in immune system development and homeostasis (2). Dysbiotic conditions (i.e. diminished GM complexity or an imbalanced GM composition), which can be caused by genetic disposition or exogenous factors such as specific diets and antibiotic use, are linked to a decreased ability to develop local and systemic immune responses (3). In mice, the absence of a gut microbiota is reflected by increased levels of arginine and proline metabolism (urea cycle), oligopeptides, carbohydrates and energy metabolism, secondary metabolites (flavonoids, phenolic acids, and terpenes), and decreased levels of bile acids, acylcarnitines, fatty amides, aromatic amino acids, lysine and polyamine metabolites, thus highlighting the broad significance of the GM to different host physiological systems (4). As for the immune system, the GM has an important impact on the growth and function of myeloid cells in many organs and at different stages of cellular development. Myeloid cell growth in the bone marrow is diminished in the absence of the GM, resulting in a delay in the clearance of systemic bacterial infection (5). The complexity of the GM also influences the level of myelopoiesis, which is modified in accordance with the presence of GM-derived Toll-like receptors (TLRs) ligands in the blood (6). Short-chain fatty acids (SCFA), the main metabolites produced by bacterial fermentation of dietary fibre in the gastrointestinal tract, may also promote myelopoiesis in the bone marrow (7). Moreover, even before birth, the microbiome has a significant impact on myelopoiesis. The offspring of antibiotic-treated mice has less circulating neutrophils (and their bone-marrow precursors), while prenatal colonization with commensal microbes increases the amount of intestinal mononuclear cells in new-born mice (8). Effector mechanisms of myeloid cells such as the formation of neutrophil extracellular traps are also influenced by the lack of a GM (9). Therefore, the central position of the GM in the holobiont is crucial to the ontogeny of the immune system, and in particular the innate immune system, with functions extending far beyond the gastrointestinal tract.

Emerging evidence in humans and mice suggests that the immunoregulatory function of the GM also pertains to distal organ sites including the airways and the brain (10, 11). Recent studies have highlighted the influence of the GM on lung immunity, referred to as the ‘gut-lung axis’, though the underlying causalities and mechanisms are still being investigated (12), including the effects of various diets on lung health and disease (13, 14). Consistent with this, adverse changes in the composition of the GM (dysbiosis) are linked to increased susceptibility to respiratory diseases and infections. Inflammatory disorders in the airways, such as asthma and chronic obstructive pulmonary disease (COPD), have been related to gut dysbiosis in humans (15, 16). In asthma, the abundance of the gut commensals *Akkermansia muciniphila* and *Faecalibacterium prausnitzii* is significantly reduced in allergic patients compared to healthy controls (17). Caloric restriction diet and supplementation with prebiotics (e.g. fructo-oligosaccharides, inulin type fructans, raffinose) have positively regulated the abundance of these commensals in mouse and human studies (18). In COPD, low fibre consumption was linked to lower lung function scores and a higher incidence of persons with airway obstruction (13). Moreover, human epidemiological studies indicate that microbial dysbiosis can have long-term consequences, which is supported by data from mouse models pointing to an increased predisposition to allergic inflammation following early life antibiotic usage (19, 20). Even if the mechanisms bridging gut microbiota with the alterations of respiratory disease outcome are still poorly understood in domestic animals, a growing body of research in poultry and swine supports a crosstalk between the gut and airways microbiome (21). The increase in the prevalence of respiratory disorders in farm and companion animals have reached epidemic statistical levels in wealthy countries that generate a greater need to monitor and control their impact on morbidity and mortality.

Altogether, it becomes increasingly clear that microbial gut colonisation and immunocompetence are interrelated and impact each other in homeostasis and/or disease. Dysbiosis and subsequent dysregulation of microbiota-related immunological processes affect the onset of the disease, its clinical characteristics, and responses to treatment. Finally, considerable attention has and continues to be paid to early-life changes to the GM and their influence on the immune system resilience to pathogens in both the gastrointestinal and respiratory mucosa. Although research on these aspects are well advanced in humans and mice, relatively little was done to interrogate these relationships in poultry, the most important segment of the livestock industry. The poultry sector, which has an extremely important place in terms of food safety and nutrition, is the fastest growing agricultural sub-sector, especially in developing countries. Some of these topics are discussed in detail in the sections below.

### Microbiota-derived short-chain fatty acids are broad immunomodulatory metabolites

The GM ferments the dietary fibre that reaches the colon in an anaerobic environment, producing short-chain fatty acids (SCFA) as metabolic by-products. SCFA play a major role in intestinal homeostasis, explaining why changes in the microbiota can contribute to the pathophysiology of human gastrointestinal diseases (22). SCFA are the most studied microbiota metabolites, possessing regulatory properties on various aspects of host physiology, including the immune system (23) and the cardiovascular system (24). The pleiotropic functions of SCFA in mice range from maintaining and reinforcing intestinal-epithelial integrity and cardiovascular homeostasis, to dampening inflammation in the gut and respiratory tract, demonstrating their importance in the local and peripheral milieu (2, 7, 10, 25). How commensal-derived SCFA participate in the crosstalk between the gut and the lungs is only starting to be unveiled (26). However, their participation is clearly complex and multifaceted, since SCFA are able to influence, directly or indirectly, the function of various cells including epithelial cells and innate and adaptive immune cells. Their biological activity is regulated by their relative availability and affinity for G-protein coupled receptors (GPCR) of the free fatty acid receptors (FFAR) family, transporter molecules, and downstream effector molecules in different cell types (27, 28).

SCFA produced by the GM in the cecum and the colon can be found in hepatic, portal, and peripheral blood. These SCFA affect the lipid, glucose, and cholesterol metabolism in various tissues, indicating that SCFA are transported from the intestinal lumen into the bloodstream and are taken up by organs where they act as substrates or signal molecules (29). The transporters for the uptake of SCFA from the blood into the tissues remain largely unknown. Overall, the concentration gradient for SCFA decreases from the terminal gut lumen (where they are abundant) to the periphery, with preferential uptake of butyrate by the intestinal epithelium, propionate by the liver and acetate by various peripheral organs (30, 31). In humans, the total SCFA concentration in the lumen of the colon decreases progressively from the proximal to the distal end from 70-140 mmol/l to 20-70 mmol/l respectively (32) with the abundance ratio of acetate, propionate and butyrate in the colon being 60:25:15 (33). Exogenous acetate formed by colonic bacterial fermentation enters the blood compartment and is mixed with endogenous acetate released by tissues and organs. Up to 70% of the acetate is taken up by the liver. To prevent high SCFA concentrations in the bloodstream, the liver also clears the major part of propionate and butyrate from the portal circulation. The major part of butyrate is used as fuel for colonocytes, the remainder is oxidized by hepatocytes within the cell or by secretion into the plasma within triglyceride-rich very low density lipoproteins, thereby preventing toxic systemic concentrations.

The main SCFA from the GM, namely acetate, propionate and butyrate, act through the activation of specific GPCR: FFAR3/GPR41, FFAR2/GPR43, GPR109A/HCAR2 and OR51E2/Olfr78 (34). FFAR2 may interact with FFAR3 to form a FFAR2-FFAR3 receptor heteromer with signalling that is distinct from the parent homomers. OR51E2 is the human orthologue of mouse Olfr78. Both FFAR2 and FFAR3 are selectively activated by SCFA from one to six carbon chain length, thus responding to both acetate, butyrate and propionate (35). GPR109A/HCAR2, the receptor for niacin/vitamin B3, recognizes mostly butyrate while OR51E2/Olfr78, the receptor for the odorant beta-ionone, recognizes mostly propionate. Signalling through FFAR receptors entails different effects depending on cell type specific receptor expression and binding of different subunits of FFAR receptors or β-arrestins. For example, butyrate can inhibit reactive oxygen species (ROS) production in neutrophils in a pertussis toxin (PTX)-sensitive manner, while acetate increases ROS production in macrophages in a PTX-insensitive manner (33). As for the mechanisms of action, SCFA play a crucial role in the regulation of inflammation and mucosal immunity by stimulating or dampening the production of cytokines (mostly *via* NFκB-dependent pathways), as well as inhibiting or facilitating the recruitment of immune cells. The relative order of potency for the suppression of NF-κB activity by SCFA was determined as butyrate>propionate>acetate in different mammalian *in vitro* systems (e.g. macrophages and endothelial cells). SCFA such as butyrate also function as histone deacetylase (HDAC) inhibitors, which can act on the epigenome through chromatin remodelling changes. The importance of butyrate’s effect on HDAC was highlighted by the demonstration that trichostatin A (TSA), a specific HDAC inhibitor, mimics many of the regulatory actions of butyrate, spanning from the expression of interleukin 8 (IL-8) and urokinase receptor to cell proliferation, apoptosis, and epithelial barrier functions (e.g. paracellular permeability and cell migration) (36, 37). SCFA also maintain mucosal homeostasis by promoting B-cell IgA production and regulating T-cell differentiation. However, SCFA receptors are not expressed in lymphocytes at significant levels, although distinctively expressed by epithelial cells, myeloid cells and endothelial cells, thus highlighting their complexity in regulating innate immunity functioning along mucosal sites (34).

There are limited reports of SCFA being detected in the mammalian lung and the lung microbiota is not known to produce SCFA, possibly due to the absence of specific substrates or fermentation in healthy individuals (10, 38, 39). It is also unknown whether the lung microbiota produces sufficient amounts of bacterial molecular motifs, such as TLRs ligands, to directly impact host airway immunity (40). A direct role for SCFA in the airways is plausible, but likely to be negligible compared to its more systemic roles. Rather, the priming effects of SCFA on immune cells should predominantly occur in the periphery, notably in the bone marrow, with a subsequent recruitment to the lungs that would ultimately contribute to lung homeostasis and immunity. Indeed, acetate and propionate have been shown as the main SCFA involved in the priming of myeloid cells that will later find a niche in the respiratory mucosa of mammals (2, 10, 25). Mechanistic actions through which GM-derived SCFA elicit their protective mechanisms against allergic airway diseases and respiratory infection were recently uncovered (7). Circulating acetate or propionate are able to modulate dendritic cell (DC) hematopoiesis and functioning in the bone marrow during Th2 cell-mediated allergic airway inflammation. Both metabolites enhance the generation of macrophage and DC progenitors, and the more differentiated, common DC progenitors in the bone marrow (7, 12, 41). These DC precursors will subsequently populate the lungs where they mature into CD11b+ DC that possess impaired allergen presentation capacity and consequently activate few Th2 effector cells (42, 43). Thus, allergic airway inflammation is dampened and goes into resolution processes. During influenza infection, SCFA only influenced the expansion of macrophage and DC progenitors subsets, while other hematopoietic progenitors were unaffected (44). Macrophage and DC progenitors differentiate into either common DC progenitors or monocytes, with Ly6C (also known as Gr-1) expression distinguishing two subsets of monocytes (45).. In inflammatory conditions, Ly6C+ monocytes can give rise to inflammatory macrophages or DCs that can cause immunopathology (45, 46). By contrast, Ly6C− monocytes can differentiate into alternatively activated macrophages (AAMs) in the lungs, which possess anti-inflammatory and tissue repair capacities (47, 48). Butyrate or propionate enhanced macrophage and DC progenitors and their differentiation into Ly6C− monocytes without affecting the Ly6C+ subset in a FFAR3-dependent manner during influenza infection. In the lungs, these Ly6C− monocytes adopted an AAM phenotype that had limited capacity to express the neutrophil chemoattractant CXCL1 (44, 49). Thus, AAMs limited the influx of neutrophils into the airways and resolved neutrophil-associated immunopathology during pulmonary viral infections (10, 44, 50).

These data illustrate that along the gut–lung axis, although context-dependent, SCFA are priming myeloid cells in the bone marrow, which subsequently migrate to the lungs and shape an anti-inflammatory milieu. Targeting this axis holds great potential for future therapies, but given the pleiotropic effects of GM-derived metabolites, and the fundamental implications of regulating the immune system at the level of both hematopoiesis and mucosal immunity, further studies are warranted, including in different biomedical models and species. Most of the work discussed was performed in mammalian model species, especially mice. Virtually no mechanistic work has been performed in detail in the major livestock species, that is chickens, pigs and cattle.

Poultry, and more specifically the domestic chicken, is the world’s primary source of animal protein (https://www.fao.org/poultry-production-products/production/en/). By 2050, the world population is expected to increase to more than 9 billion people and the demand for animal protein will at least double. Providing food for this growing human population while respecting the balance between animal welfare, quality products, consumer acceptance and safety, is a major priority in the UN Sustainable Development Agenda, targeted in its “Zero Hunger” goal (https://www.un.org/sustainabledevelopment/hunger/). Attempting to control or influence microbial colonisation patterns of the young chicken’s gut (including during embryonic development) to promote health and productivity has today become a focus in modern poultry production (51, 52). In order to develop novel solutions to poultry health issues, including novel vaccines and/or vaccine adjuvants, and the identification of disease resistance genes which can inform breeding programmes, it is necessary to gain a more thorough understanding of the avian immune response and how pathogens can subvert that response. Likewise, mechanisms at the interface of the dialogue between the GM and the mucosal immune system in poultry remain undefined. Birds and mammals share the same environments, have similar lifespans and body masses, and confront similar disease threats, yet birds have a distinct immune system repertoire than mammals, with different organs, cells, molecules, and genes (53). The immune system of avian species, such as chickens, is recognized as being very distinct from those of model mammalian species. For example, unique features such as caecal tonsils (the largest lymphoid aggregates in the bird’s gut) and the bursa of Fabricius (a B-cell powerhouse), both located next to the distal regions of the gastrointestinal tract, makes the avian gut-associated immune system a very particular anatomical and immunological landscape compared to mammals. Extrapolation from mammalian systems that has not been well tested cannot give the level of information needed to comprehend microbiota-host-pathogen relationships. Therefore, we are still some way from a clear understanding of the interactions between the GM and immune system components, and the specific interventions that would promote chicken health and productive performance in environmental responsible livestock farming. Understanding gut health has been a primary focus of the poultry industry worldwide as a means of increasing production of meat and eggs, reducing the use of antibiotics, and enhancing animal welfare. The poultry industry has been at the forefront of advances in the development of pre- and probiotics, nutritional antioxidants, essential oils, anti-nutritional enzymes, and immune modulators for the regulation of gut health and functionality (54).

### Microbiota acquisition in poultry

The gastrointestinal compartment of chickens are densely populated with a complex GM that are dominated by bacteria (55, 56), as observed for most vertebrate species. The interactions between the host and the chicken GM have been extensively studied (57–61) and are now established as playing important roles in bird nutrition, physiology and gut development (62, 63). Furthermore, the immediate rearing environment profoundly influences the development of the chicken GM (64). Chicks acquire their GM under natural rearing conditions from the eggshell and/or by ingesting faecal bacteria from adult hens (‘maternal flora’). However, in modern poultry hatcheries (with egg surface disinfection and absence of chick-hen contacts) this GM transfer is hampered, with potentially negative consequences for animal health and welfare. Colonisation of commercially hatched chicks is therefore exclusively dependent on environmental sources, during which the animals’ caeca are first colonised by *Enterobacteriaceae* (phylum *Proteobacteria*), which become replaced by *Lachnospiraceae* and *Ruminococcaceae* (phylum *Firmicutes*) during the second week of life (64). At around four weeks of life, *Firmicutes* are joined by bacterial isolates belonging to the phylum *Bacteroidetes* (65). This gradual GM development during the first weeks of life may render chicks highly susceptible to pathogens targeting the gastrointestinal tract, such as *Salmonella* (66, 67). In turn, it is well established that inoculation of chicks with the GM from adult hens can increase their resistance to *Salmonella* (66, 68, 69). Despite the importance of these observations, studies focused on strategies to promote GM transfer between hens and chicks - or the early life implantation of a complex commensal microbiome - in commercial poultry are scarce.

It is unclear if all GM members or just a small subset of them are successfully passed from hens to chicks. It is also unclear how fast GM transmission between hens and chicks occurs, or whether contact between the hen and the chicks must continue for days or weeks. Nevertheless, GM transfer is supposed to be quick, since 24 hours-long contact between a donor hen and its chicks appeared to be enough for successful transfer (64). *Bacteroidetes*, *Actinobacteria*, *Selenomonadales* and *Faecalibacterium* are all efficiently transferred from donor hens to chicks. However, the transfer of *Lactobacilli* or *Clostridiales* is often not observed (64). These observations should be considered when designing the next generation of probiotics or when performing faecal microbiota transplantations as tested earlier (64, 65). Although *Lactobacilli* or *Clostridiales* may have an influence on the development of the gastrointestinal tract just by passing through it, the beneficial effect of probiotics on gut health will most likely prevail when probiotic bacteria are able to implant and colonize the gut successfully.

**Figure 1:**
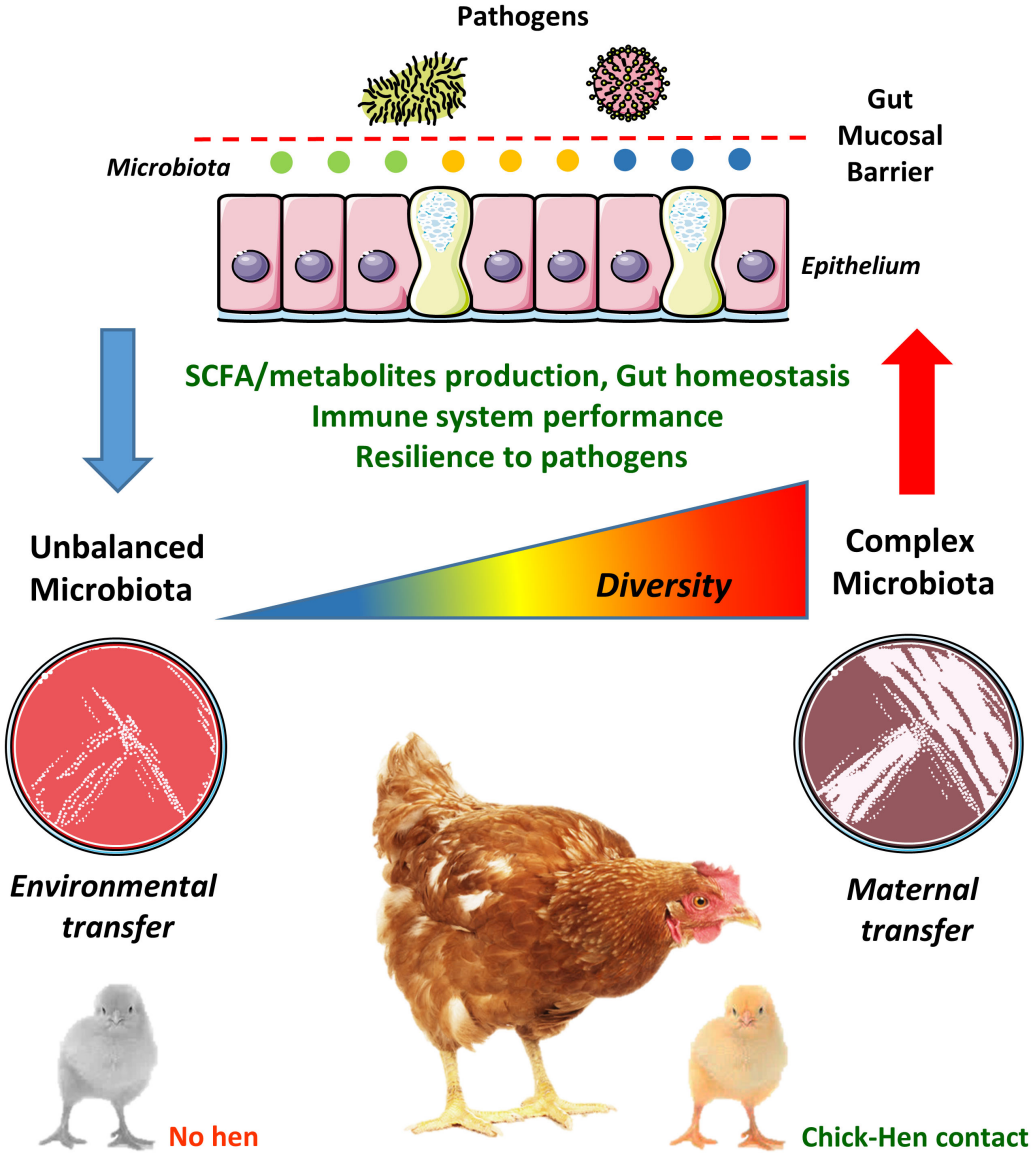
Chick-hen contact influences microbiota acquisition and diversity in early life. The gut microbiota (GM) is usually transmitted vertically from mothers to newborns, where pioneer colonizers (i.e. the first bacteria reaching the neonatal gut) greatly influence the development of a complex microbiome. However, the current commercial poultry production systems, with its high-standard hygiene procedures, has eliminated any contact between hens and chicks. Therefore, chicks are colonized by microbes present in the environment, feed and transferred by human contact, which often hampers the establishment of a diverse and well-balanced GM in early life. This may lead to negative consequences to gut physiology and homeostasis, such as altered short-chain fatty acids (SCFA) and metabolites production, dysfunctional gut mucosal barrier, reduced immune system performance and increased susceptibility to pathogens. These alterations are probably not restricted to the gut environment and could impact remote mucosal sites such as the lungs (the gut-lung axis).

Given its biological and economic implications, it is surprising that GM transfer between hens and chicks has not been studied in more depth, especially given the recent advances in next-generation sequencing which now allows past technical challenges to be readily addressed. The 16S rRNA gene is commonly used to identify and compare bacteria present in a given sample (70). Accessible bacterial databases, such as Greengenes (71) and Silva (72), in addition to well-developed bioinformatics pipelines are available to facilitate these analyses (73–75). Therefore, the knowledge gained can be utilized not only to identify bacterial species that are efficiently transferred from hens to chicks but also to isolate them in pure culture. Pure cultures of such isolates or combinations thereof should thus imitate the natural transmission from hens to chicks, improving gut health in the chicks from the first days of life. This would also allow a thorough scrutinisation of the individual contribution of selected pioneer bacteria in the development of key physiological systems, such as the immune system, thus facilitating the development of novel and more efficient probiotics. Therefore, only an evidence-based method relying on the principles of natural GM transfer between the hen and the chick would allow the development of improved poultry production practices aiming to increase poultry performance and robustness in early life (64). Recently, a study showed that GM transfer in early life is possible even between different commercial chicken lines (76), revealing that progress is being made towards a wider utilization of the GM transfer concept in commercial poultry. The relationships between early hen-chick contact and GM complexity in the development of the gut mucosal barrier is illustrated in **Figure 1**.

As for the lung microbiome, research in poultry species is still in its infancy. Recent studies attempting to characterize the respiratory microbiome of poultry have focused primarily on bacteria, as there are well established and rapid methods of sequencing and analyzing this component in the lungs of livestock species (77–82). Glendinning and colleagues performed 16S rRNA gene sequencing to characterize the nasal and lung microbiota of chickens (80), showing that members of the genus *Lactobacillus* are by far the most common bacteria identified in swab samples. Utilizing similar methods, Shabbir et al. determined that the lower respiratory tract of healthy chicken flocks from different farms in Pakistan exhibited high levels of diversity in their microbiota (82). More recently, Johnson et al. presented a comprehensive analysis of the core bacterial microbiota in the broiler gastrointestinal and respiratory tracts and barn environments (81). Although *Lactobacillaceae* were the most common bacteria in the trachea and in the ileum, the leading *Lactobacillus* species in these tissues had different relative abundances. Despite the fact that the bacterial component gives useful information on the respiratory microbiome, a complete analysis of the avian respiratory microbiota has yet to be published, and it is still underappreciated when compared to research addressing the complexity of the GM (83). Correlations between lung microbiota and immunity, including the potential contribution of GM transfer between hens and chicks, are therefore largely inexistent. The mechanisms underlying those potential interactions are virtually impossible to be predicted at present. Broilers and layer hens are subjected to rigorous vaccination programs, with the most common vaccine methods being spray or eye/nose drop applications, thus highlighting the central role of mucosal surfaces in poultry vaccination. Vaccines administered by spray or aerosol are licensed worldwide for utilization against several poultry diseases such as infectious bronchitis, *Mycoplasma gallisepticum* infection, Newcastle disease, avian rhinotracheitis (pneumovirus) and coccidiosis (droplets are ingested rather than inhaled). Changes in the composition of the respiratory microbiota in mammals have been shown to be correlated with various respiratory diseases and to vaccination against specific respiratory pathogens (84, 85). Therefore, further studies in this area are warranted in chickens and other commercial poultry.

### Short chain fatty acids in poultry and their regulatory functions

As in mammals, SCFA are very abundant in the chicken gastrointestinal tract, where their concentrations (in the millimolar range) vary depending on the diet, host genetics, breed conditions and notably GM composition (86–88). The pH values, as well as the concentration and proportion of SCFA and lactate, change along the chicken gastrointestinal tract due to the sites of fermentation and in an age-dependent manner, and are therefore often regarded as good parameters to follow an adequate development of the gut. The caeca contain the highest amount of SCFA and lactate at the first 6 weeks of age. In 2 weeks-old old birds, the percentages of lactate: acetate: propionate: butyrate in the caeca is 49: 37: 11: 3. However, it changes to 12: 73: 5: 11 from 4 weeks onwards. The caeca, probably due to their larger and diverse microbial population and longer transit time of the digesta (89) had higher SCFA concentrations compared with the small intestine (e.g. ileum). Previous studies on pigs (90) and chickens (91) already reported elevated concentrations of acetate, propionate and butyrate in the distal parts of the gastrointestinal tract. Rehman and colleagues hypothesized that the lower amounts of SCFA in the proximal parts of the GIT might be due to the lower bacterial metabolic activity and short transit digesta time in this part compared with the caeca (91). Therefore, the concentrations of SCFA in chickens are overall similar to those observed in humans and mice, suggesting that their regulatory actions at the gut epithelia are probably similarly in place, and that they could eventually leave the gut and act at distant organ sites. However, to our knowledge, their presence outside the gut of chickens or other poultry species was not yet evaluated.

The addition of the SCFA butyrate in the form of sodium butyrate has been seen as an alternative to promote the chicken’s gastrointestinal development and overall zootechnical performances (92, 93). This compound is solid, stable and is associated with the improvement of body weight, feed conversion ratio and the development of the gut mucosa by increasing villus height and crypt depth ratio in the duodenum and the jejunum (94–96). Regarding gut intestinal microbiota, butyrate is associated with increased beneficial bacterial populations, such as *Lactobacilli* and *Bifidobacteria* while colonization with potentially pathogenic species is reduced (96). Indigestible non-starch polysaccharides (NSP) in the feed serve as substrates for bacterial fermentation, greatly contributing to the production of SCFA. NSP represent a major part of the dietary fibre component in plant-based feed ingredients, accounting for approximately 10% of the nutrients in the average poultry diet. However, NSP are generally not considered during formulation of commercial broiler diets. Borda-Molina and colleagues recently indicated that the composition of the diet is the main factor affecting the overall structure of broilers’ GM and consequently SCFA production, where the source of NSP as a substrate for bacterial fermentation had a stronger stimulus on bacterial communities than crude protein content or direct supplementation of sodium butyrate (97).

Nevertheless, data available for the beneficial effects of sodium butyrate on chicken performance and health are not always consistent. Firstly, butyric acid can be administered either in its free form, associated with a salt, and/or protected in a glyceride matrix. Secondly, it may be supplemented at different doses and/or under different experimental conditions (e.g. chicken breed, trial duration, presence or absence of specific challenges and stress conditions). Differences among studies may be also related to the hygiene status of the environment to which chickens are exposed. Several authors have already reported that there is no growth-promoting response to fat-coated butyric acid (or its mono- and diglycerides) when chickens are reared in an specific pathogen-free (SPF) environment or where the overall health status is good (94, 98), although the physiological mechanisms underlying these observations were not detailed. In summary, under good health conditions, the current consensus is that sodium butyrate would have no major effects on poultry performance and gut morphology at different ages, at least for broiler chickens (99). The studies on butyrate underscore that extensive research of SCFA metabolism, biological functions and stability metabolism are needed to provide a solid rationale for the utilization of SCFA in commercial poultry farming.

The ability of SCFA to modulate inflammation and promote homeostasis was also assessed in livestock species, with butyrate being by far the most studied SCFA. In poultry, supplementation of butyrate in the feed benefits immune responses in the chicken, including by limiting the concentrations of pro-inflammatory cytokines and corticosterone, the latter being abundant during stress conditions (87, 94, 95). At the cellular level, a study showed that SCFA play distinct roles in the activation of intestinal regulatory T cells (Tregs) (100), although, avian regulatory T cell research is still poorly standardized (101). Another study by Sunkara and colleagues suggested that butyrate induces the expression of antimicrobial peptides in chicken macrophages (102). The same authors also revealed that acetate, propionate, and butyrate exert a strong synergy in augmenting antimicrobial peptides gene expression in chicken primary macrophages and a macrophage cell line (103). Butyrate has also been shown to reduce *Salmonella* Enteritidis colonization and shedding in chickens, as well as limiting the appearance of necrotic lesions induced by *C. perfringens* in the small intestine (104, 105). Nevertheless, mechanistic data on the immunoregulatory functions of SCFA other than butyrate are scarce in poultry, although a broader understanding of their biological functions in the chicken gut has seen some progress in recent years (106).

Several receptors for butyrate have been identified in human and mice, including FFAR3, FFAR2, and GPR109α (107). As discussed earlier, FFAR2 is capable of recognizing not only butyrate but also acetate and propionate (108). However, data on the presence and function of these receptors in chicken tissues or cells are very limited. For example, it is well established in mammals that the activation of FFAR2 by SCFA promotes the number and function of IL-10+ Foxp3+ regulatory T cells (Tregs), where propionate can directly increase Foxp3 expression and IL-10 production by Tregs (109). In addition, both butyrate and propionate are known to induce the differentiation of Foxp3+ Tregs. In the chicken, however, only acetate, but not propionate or butyrate, is able to promote the expansion of CD4+CD8−CD25+ and CD4+CD8+CD25+ Tregs in caecal tonsils (the largest lymphoid aggregates of the avian gut-associated lymphoid tissue) (100). Moreover, FFAR2 was shown to be highly expressed in CD4+CD8−CD25+ Tregs. How CD4+CD8−CD25+ T cells were affected by acetate is not clear, as the role of FFAR2 expression in the regulatory function of T cells has been controversial (25, 109), especially in less studied species such as chickens. Importantly, genes encoding GPR109A and FFAR3, other key SCFA receptors, are not found in the chicken genome (110, 111). Therefore, the physiology and pharmacology of GPCR receptors regulating SCFA functions in chicken immune cells remain largely undefined.

It was previously demonstrated that more than 20 genes encoding FFAR2 paralogs exist in the chicken genome (111). Although all FFAR2 paralogs seem to encode full-length proteins, the functionality of those genes remains unknown. Moreover, this expansion among FFAR2 genes seems to be chicken-specific, as it was not found in other galliform birds such as turkeys and quails. Interestingly, the same authors highlight that massive duplication of FFAR2 can be found in an ancestral chicken line from Thailand, therefore dating this gene expansion event between 30 Ma (estimated divergence between chicken and turkey) and 8,000 years ago (chicken domestication). Since chickens in modern poultry production systems are usually fed cereal-based diets without major sources of fibres, the physiological significance of this large FFAR2 duplication remains unresolved. In humans, specific duplications of amylase gene were described, and copy number of this gene was shown to be positively correlated with salivary amylase protein level (112). Moreover, individuals from populations with high-starch diets have more copies than those with traditionally low-starch diets. Therefore, an adaptation to diet in domesticated poultry is the most reasonable hypothesis to explain numerous FFAR2 gene duplications. In humans and mice, FFAR2 has been shown to mediate the beneficial effects of high soluble fibre diets in limiting the pathological manifestations of obesity and dyslipidemia (113). Activation of FFAR2 by SCFA also leads to the inhibition of lipolysis and the decrease of free fatty acids levels in the serum (114), a phenomenon that could be amplified by the presence of these numerous FFAR2 paralogs in the chicken, although not yet experimentally tested.

Overall, the striking FFAR2 gene expansion and the potential complexity of SCFA signalling in the chicken stresses the need for further assessing the intricate dialogue between the GM and its regulatory metabolites within the epithelial and myeloid landscape along the gut-lung axis.

### Microbiota, immunity and infection in poultry: The gut-lung axis revealed

Modern selection programs implemented by industrial chicken breeders favour high performance traits, such as rapid broiler growth, prolific egg production and efficient feed conversion. However, selection for a single trait may also affect other traits, with potentially negative effects on the GM and immune system development, as already reviewed or reported elsewhere (52, 115, 116). These so-called indirect effects of “selection for performance” in poultry physiology are continuously being investigated by the poultry research community worldwide. Moreover, as discussed above, GM development in commercial poultry is minimized by the absence of contact between chicks and hens. Disruption of the GM composition and balance in modern poultry is often recognized as a consequence of the aforementioned poultry production practices in conjunction with subclinical pathologies commonly observed in poultry flocks (116). Today, dysbiosis is scarcely used as a read-out parameter for assessing animal health and hygiene status in poultry flocks – although it can severely impair gastrointestinal and immune system development and functioning in young birds (52), thereby favouring the occurrence and/or severity of infections/superinfections.

Viruses are among the leading causes of animal and production losses in both commercial flocks and backyard chickens. Contrary to bacteria, which can be treated with antibiotics, there is no drug therapy available for poultry to treat viruses. Supportive measures are applied as part of the treatment. Vaccination (when available), biosecurity measures, or even culling of the whole flock are used to prevent and/or control the disease. Antibiotics may help to mitigate the severity of an outbreak if a bacterial superinfection is present, such as Avian Colibacillosis (117), which often occurs in respiratory infections such as Newcastle Disease, Infectious Laryngotracheitis or Avian Influenza. Therefore, the relevance of supporting a robust GM and immune system development along the gut-lung axis in early life is still one of the best strategies to promote health, welfare and to limit economical losses due to viral infections in poultry flocks.

As for the relation between viral infections and the GM, influenza virus infection (flu) has been shown to cause dysbiosis in mammalian species (via type I interferon-mediated effects on the GM composition) (118). Avian influenza viruses (AIV) are highly contagious and highly variable viruses that infect birds in large numbers. Domesticated poultry and other birds can be infected, but wild birds in aquatic habitats are thought to be the natural reservoir hosts, thus posing a threat to chickens under free-range or organic breeding conditions. Low pathogenic avian influenza viruses (LPAIV) are viruses that cause only minor disease in poultry. Certain LPAIV can evolve into highly pathogenic avian influenza viruses (HPAIV), which are most commonly found in poultry flocks (119). HPAIV can kill up to 90% of a chicken flock, causing epidemics that can devastate the poultry industry and lead to trade restrictions. The presence of LPAIV that can evolve into HPAIV in poultry has the potential to disrupt international trade. Although virtually invisible compared to HPAIV, viral infections caused by LPAIV are relatively prevalent in European poultry flocks (120) and some strains are known to infect humans (e.g. H7 and H9 subtypes). Although LPAIV may favour severe superinfections (bacterial, fungal), LPAIV itself generally cause mild or subclinical disease and have limited impact on poultry performance.

In contrast to human influenza viruses, that replicate in the respiratory tract, AIV also replicate in gut epithelial cells and can be transmitted *via* the faecal-oral route (121). The relevance of infection by avian influenza viruses to the gut-lung axis concept is therefore of prime relevance. In the gastrointestinal passage, the virus is exposed to acidic fluids of the chicken’s gastrointestinal tract that are rich in the proteases pepsin (gizzard) and chymotrypsin/trypsin (intestine). The latter enzyme render the virus active in the avian gut through hemagglutinin (HA) cleavage into HA1 and HA2 subunits. However, due to a lack of a suitable cell culture system, little is known about whether epithelial cells of the avian gut release infectious virus particles and cleave HA with a monobasic and/or polybasic cleavage site (121). Therefore, AIV-induced dysbiosis in early life (due to direct, local and/or type-I IFN-mediated remote effects) could impair proper maturation of the young chicken immune system. On the other hand, dysbiosis due to an unbalanced GM composition and altered immune system development and functioning could favour respiratory and/or intestinal AIV infection with increased severity and negative consequences to the poultry production sector. This two-way dialogue was never addressed in poultry, although Chrzastek and colleagues confirmed that LPAIV infection retards caecal microbiota diversification in the chicken (122). Moreover, Yitbarek and colleagues already suggested that shifts in the composition of the GM may result in changes in cell- and antibody-mediated immune responses to vaccination against avian influenza viruses (123). Therefore, enhancing immunity along the gut lung-axis *via* increased GM diversity in early life are among the best strategies to foster resilience to economically important poultry viral pathogens, such as AIV. This hypothesis was tested in parts, with new experimental evidence showing that chick-hen contact in early life improves microbiota stability and host response to infection with a H9N2 LPAIV strain (124).

**Figure 2:**
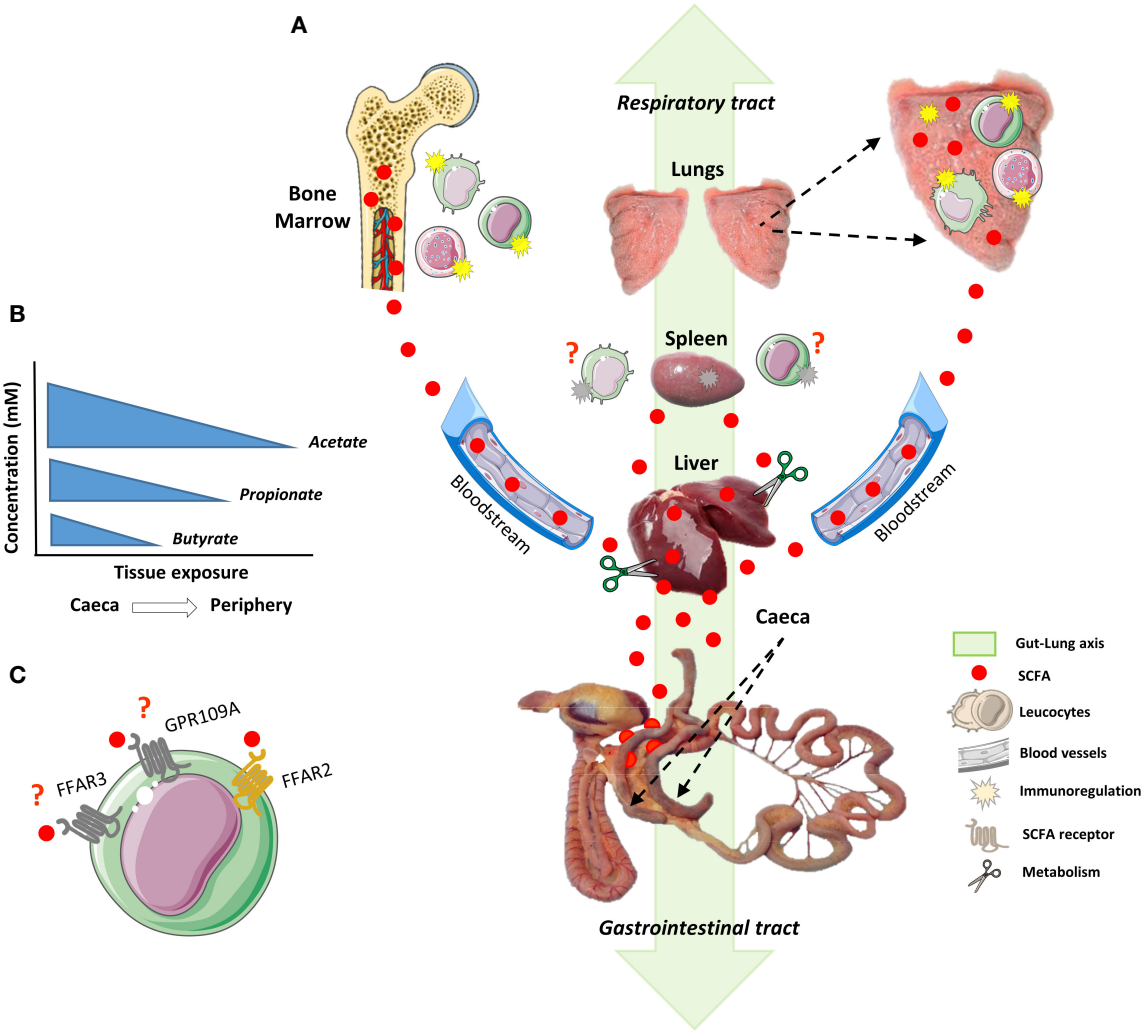
The gut-lung axis and how it may operate in the chicken. The development of the immune system goes hand in hand with the acquisition and maintenance of a complex gut microbiota (GM). The GM promotes and calibrates multiple aspects of the immune system maturation and functioning *via* the release and production of various molecular motifs and metabolites, respectively. These molecules not only modulate local (gut) immunity, but also exercise remote functions on peripheral organs, including the lungs: a phenomenon referred to as the gut–lung axis. In chickens, its existence and functioning remains elusive. **(A)** Large amounts of short-chain fatty acids (SCFA) are produced in the caeca, a microbiota-enriched portion of the chicken gut where fermentation of dietary polysaccharides takes place. SCFA, the main bioactive end products of fermentation can then act locally (in the gut), or leave the gastrointestinal tract through the bloodstream. When taken up, a large part of the SCFA is used as a source of energy. The part of SCFA that is not consumed by the caecal epithelial cells is transported across the basolateral membrane. To prevent high SCFA concentrations in blood, the liver clears the major part of acetate, propionate and butyrate from the portal circulation. As a general rule it is believed for humans, rodents and livestock species alike, that SCFA concentrations are regulated through a preferential uptake of butyrate by the intestinal epithelium, propionate by the liver and acetate by various peripheral organs **(B)**. The remainder of SCFA that may reach peripheral organs beyond the liver is mostly unknown. However, part of these SCFA could reach the bone marrow *via* the bloodstream where they play a role in the development and priming of immune cells. The SCFA reaching the lungs could act in reinforcing the epithelial mucosa or in the priming of resident immune cells, such as phagocytes. However, the role of SCFA in the spleen, the main secondary lymphoid organ, is largely unexplored. **(C)** SCFA exert their biological functions mainly through G-protein coupled receptors (GPCR), such as FFAR2, FFAR3 and GPR109A. In the chicken, FFAR3 and GPR109A are lacking, and FFAR2 has been shown to possess more than 20 paralogs. To date, the pharmacology of SCFA receptors and their mechanisms of action in the chicken are unresolved.

The immune system of birds differs from that of mammals to a great extent, especially in respect to organs located next to the terminal portion of the chicken gut, such as caecal tonsils and the bursa of Fabricius, both greatly involved in the regulation of B and T-cells ontogeny and function (53). The level of knowledge for the phenotype and function of epithelial cells and leukocytes in birds does not yet match the level achieved for humans and mice. Likewise, to add another layer of complexity to the rational exploitation of the gut lung-axis in chickens, the avian parabronchial lung substantially differs morphologically from the mammalian bronchioalveolar lung, as brilliantly reviewed by Reese and colleagues (125). The avian lung is a flow-through system, relying on a set of nine flexible air sacs that act like bellows to move air through the almost completely rigid lungs. Air moving through bird lungs is largely fresh air and has a higher oxygen content. Gas exchange takes place in the parabronchial tissue mantle, which is the fundamental functional part of the bird lung. Organized lymphoid structures in the chicken lung mucosa were found to be highly similar to Peyer’s patches and other gut-associated lymphoid tissues (GALT) and are, in analogy to mammals, designated bronchus-associated lymphoid tissues (BALT). BALT structures in adult birds consist of lymphocyte aggregates which are covered by a distinct layer of epithelial cells harbouring considerable numbers of lymphocytes. Macrophages, dendritic cells-like phagocytes and heterophils are distributed throughout the BALT nodules. More recently, the lymphoid aggregates in the whole avian lung could be visualised *via* whole mount microscopy (126). This was accomplished by the recent development and use of chickens expressing a reporter gene (eGFP or mApple) under the control of the CSF1R promoter and enhancer in cells of the mononuclear phagocyte lineage. Mononuclear phagocytes are located at strategic check points where fresh air is distributed into the gas exchange areas, allowing particles to be phagocytized and removed. In more recent studies, respiratory mononuclear phagocytes in the parabronchial mantle have been described as CD11+, MRC1L-B+ and DEC205+ for cells located in the interstitial tissue of the primary bronchus wall, and CD11+ and MRC1L-B+ for cells located in the interatrial septa (125, 126). Nevertheless, in depth characterization of the functions of these cells is still lacking. Further developments in the generation of CRISPR/Cas9-edited and reporter chicken lines (126, 127) for the identification of novel immune cell markers and the subsequent development of more specific antibodies will pave the way to a better understanding of how the mucosal immune system operates in the chicken respiratory tract, including how resident immune cells are connected to the periphery, especially in regard to the bone-marrow and the GM-derived regulatory molecules such as SCFA. A graphical illustration of our present concept of how the gut-lung axis could operate in chickens is proposed in the **Figure 2**.

In line with such advances in creating original chicken lines is the wider utilization of germ-free chickens to study the GM with reduced experimental biases (such as the utilization of antibiotics). We and others have contributed to the development of germ-free chicken models, including from the commercial fast growing broiler line Ross PM3 (128). Originally developed for an experimental White leghorn line, this protocol has been adapted not only to the Ross PM3 broiler line but also to quails (129). Besides the use of this model to investigate host-microbiota mutual interactions in the poultry gut, it could also be useful for applied research, for example to assess safety and efficacy of probiotics or bacterial preparations using chicken gut commensal microorganisms. More importantly, the application of the current germ-free hatching and rearing protocols to raise CRISPR/Cas9-edited and/or reporter chickens lines lacking a GM from hatching will revolutionize our understanding on the development and functioning of the gut-lung axis in poultry.

### The functional existence of a gut-lung axis in the chicken: Preliminary evidence

**Figure 3:**
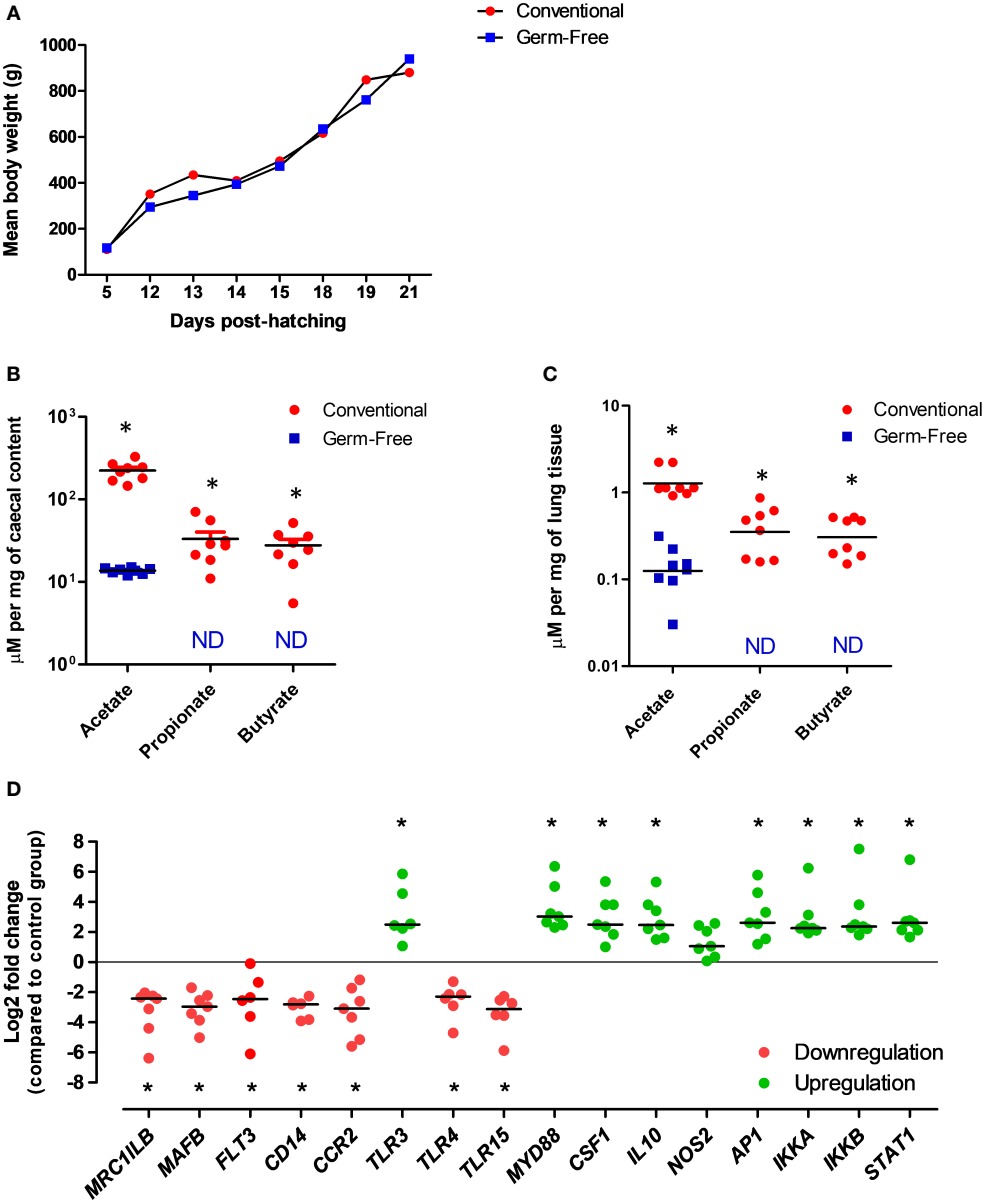
Evidences for the existence of a functional gut-lung axis in the chicken. **(A)** Germ-free and conventional inbred White leghorn chickens raised in isolators possess no differences in weight gain during the first 3 weeks of life, suggesting no major physiological anomalies. **(B)** Concentrations of acetate, propionate and butyrate in the caecal contents of conventional and germ-free chickens at 21 days of age as quantified by ^1^H-NMR. Although acetate can still be detected, possibly from the diet, propionate and butyrate are absent from germ-free caecal contents. **(C)** Concentrations of acetate, propionate and butyrate in the lungs of conventional and germ-free chickens at 21 days of age as quantified by ^1^H-NMR. Although residual concentrations of acetate are present, propionate and butyrate are undetectable in the lungs of germ-free chickens. Concentrations are shown in µM per mg of caecal content or tissue. ND, not detected (or below the threshold). **(D)** qRT-PCR analysis revealed changes in the expression of selected immune-related genes in the lungs of germ-free chickens at 21 days of age. Relative normalized expression was calculated using the 2^−ΔΔCt^ method and data are represented as Log2 fold change as compared to the conventional chickens group. Values are the median. *P < 0.05. All experimental protocols and primer pairs used for the qRT-PCR analysis are shown in the Supplementary Material.

Using the germ-free chicken model, we were already able to validate, for the first time, the functional existence of a gut-lung axis in an inbred White leghorn chicken line (**Figure 3**). Conventional or germ-free birds were sacrificed at 3 weeks of age [when immunocompetence can be reached with the decrease of maternal antibodies (130)] and lungs were recovered for the quantification of SCFA and for assessing the expression of selected innate immune genes (Supplementary Materials) between the two conditions. Germ-free chickens presented a growth rate similar to conventional animals raised under the same housing conditions and same type of diet (**Figure 3A**). As expected, conventional animals presented average concentrations of SCFA in their caecal contents, as measured by proton nuclear magnetic resonance (^1^H-NMR). However, most SCFA are undetectable in the caecal contents of germ-free animals (**Figure 3B**). The only exception is acetate, which is reduced to half of the concentration found in conventional animals. The presence of acetate is believed to be of dietary origin since sterility tests revealed the complete absence of bacteria in germ-free animals. Interestingly, we identified for the first time the presence of SCFA in the lung tissue of conventional chickens (**Figure 3C**), although in concentrations significantly lower as compared to those found in caecal contents. These metabolites are absent (or below detection threshold) in the lungs of germ-free chickens, therefore validating our hypothesis that metabolites from bacterial fermentation, possibly from the GM (since healthy lungs will harbour no fermentation processes), may reach peripheral organs. As for the technique employed, ^1^H-NMR is a highly automatable and reproducible technique for the detection of tissue metabolites, making high-throughput large-scale metabolomics studies much more feasible than with LC-MS or GC-MS (131). It requires little or no chromatographic separation, sample treatment, or chemical derivatization, allowing the routine identification of novel compounds in less well studied species, such as the chicken.

Finally, we carried out a gene expression analysis in lung samples from conventional and germ-free animals to test the hypothesis that the GM and its metabolites may regulate immune mechanisms at the respiratory mucosa. Targeting selected genes related to immune cell development and function, as well as pathogen detection, we observed that different patterns of gene expression can be observed in the lungs in the absence of a GM (**Figure 3D**). Certain genes linked to mononuclear phagocytic cell functions (*MRCL1B* – a *bona fide* marker of avian mononuclear phagocytes, *MAFB-* involved in macrophage differentiation, *FLT3 -* important for the normal development of haematopoietic stem cells and progenitor cells, *CD14 –* a macrophage-dominant PRR*, CCR2* - a CC chemokine which specifically mediates monocyte chemotaxis), bacterial (*TLR4 -* recognizing lipopolysaccharide from gram-negative bacteria) and yeast (*TLR15* - only identified in avian and reptilian genomes) molecular pattern recognition are significantly downregulated in the lungs of germ-free chickens. On the other hand, genes linked to viral molecular pattern recognition (*TLR3* - recognizing double-stranded RNA in endosomes*)*, key immune transcription factors *(AP1* - regulates gene expression in response to cytokines, growth factors and infections*, IKKA* and *IKKB –* forming a complex that plays an important role in regulating the NF-κB transcription factor*, STAT1 –* key transcription factor downstream interferon signaling), regulatory cytokines (*CSF1 -* controlling hematopoietic stem cells to differentiate into mononuclear phagocytes*, IL10 –* an anti-inflammatory cytokine), and the gene coding for the universal TLR adapter protein MyD88 (*MYD88*) are significantly upregulated (**Figure 3D**). These data indicate that at least cells of the mononuclear phagocyte system and TLRs in the respiratory mucosa of chickens are likely to be regulated by molecular signals coming from the GM. Although the biological significance of these findings remains undefined, this preliminary gene expression profiling corroborates our metabolomics data in the sense of supporting the functional existence of a gut-lung axis in chickens. Further high-throughput analysis (e.g. RNA-seq, scRNA-seq) coupled to flow cytometry and immune-histological techniques will help define the contribution of SCFA to immune cells maturation and functioning within the chicken respiratory mucosa.

## Concluding remarks

Over the past decade, the field of immunology has been revolutionized by the growing understanding of the fundamental role of the GM in the induction, training and function of the immune system. The highly regulatory tone of the neonate immune system and the action of early-life gut commensals in the development and training of this system lead to the establishment of a durable and homeostatic host/commensal relationship beyond the gastrointestinal tract, which led to the discovery of very complex physiological dialogues such as the gut-lung axis. SCFA, key metabolites involved in the regulatory functions of the GM within the gut and the peripheral system, has been show as pivotal signaling molecules in bridging gut health and resilience to pathogens in both the respiratory and gastrointestinal tracts. Nevertheless, the importance of early gut colonization for the chicken was largely neglected despite the particular situation in modern poultry farming where chicks are prevented from acquiring a well-balanced and essentially healthy GM from the hen. Moreover, only recently new technologies and tools have been developed and used for a better understanding of the particularities of the chicken mucosal immune system. Thus, it is becoming increasingly clear that in-depth analyses of the impact of the GM on immune system development and resilience to environmental stressors and pathogenic challenge will generate new knowledge and pave the way for the development of novel GM modulation strategies (e.g. GM transfer and tailored feed formulations) to be applied in poultry farming, thereby mutually benefitting animal health and welfare and consumer safety. Our data presented here point to the existence of a functional gut-lung axis in chickens, which must now be further explored in greater details. In a nutshell, the gut-lung axis starts to become a fully exploitable concept in the field of poultry sciences.

## Data availability statement

The original contributions presented in the study are included in the article/**Supplementary Material**. Further inquiries can be directed to the corresponding author.

## Ethics statement

The animal study was reviewed and approved by the French regional ethics committee number 19 (Comité d’Ethique en Expérimentation Animale Val de Loire) under the reference APAFIS#30655-2021042114338110 v1.

## Author contributions

RG conceived the hypothesis, designed the study, performed the experiments, analysed data, acquired funding and wrote the manuscript. VS-M performed the experiments and revised the manuscript. PQ helped conceive the hypothesis and revised the manuscript. ST helped conceive the hypothesis, wrote the manuscript and acquired funding. All authors contributed to the article and approved the submitted version.

## Funding

Work presented here is funded by INRAE, France (Santé Animale – SA and Microbiologie et Chaine Alimentaire – MICA), Région Centre-Val-de-Loire, France (“INTEGRITY” and FEDER “EURO-Féri”) and VetBioNet, EU H2020 (Grant Agreement N° 731014).

## Acknowledgments

We thank Vanaique Guillory (ISP, INRAE), the staff from the Plate-forme d’Infectiologie Expérimentale (PFIE, INRAE), and our colleagues from the Centre International de Ressources Microbiennes - Bactéries Pathogènes (CIRM-BP, ISP, INRAE) for providing technical support, animals and sterility checks, respectively.

## Conflict of interest

The authors declare that the research was conducted in the absence of any commercial or financial relationships that could be construed as a potential conflict of interest.

## Publisher’s note

All claims expressed in this article are solely those of the authors and do not necessarily represent those of their affiliated organizations, or those of the publisher, the editors and the reviewers. Any product that may be evaluated in this article, or claim that may be made by its manufacturer, is not guaranteed or endorsed by the publisher.
