# Supplementary material for: Uncovering the core principles of the gut-lung axis to enhance innate immunity in the chicken
Source: Front Immunol. 2022 Oct 4;13:956670. doi: 10.3389/fimmu.2022.956670 (PMC9577073; doi:10.3389/fimmu.2022.956670)
Supplement: Supplementary file 1 [file DataSheet_1.pdf]

Supplementary Material

1. Material and Methods

1.1. Animals

Germ-free chickens were reared using a protocol previously described (1). Briefly, eggs of an inbred white leghorn layer chicken line (PA12) were obtained from specific pathogen free (SPF) hens kept at standard breeding conditions (INRAE, Plateforme d'Infectiologie Experimental, PFIE, Nouzilly, France). Clean and flawless eggs were disinfected and incubated in a sterile hatching incubator. Upon hatching, germ-free chicks were transferred to sterile isolators under BSL2 containment. Eggs used to generate conventional birds were hatched under SPF conditions without any specific disinfection procedure. At day 1 post-hatching they were placed in similar isolators as for germ-free animals. Birds were fed ad libitum with a commercial diet (Safe 115, Safe-Diets, France). Germ-free birds received the same feed sterilized by gamma irradiation. Tap water (autoclaved for germ-free birds) was provided by water dispensers. Birds were kept under the conditions described above for 21 days. Sterility tests were performed routinely in pooled faecal contents from birds within a given isolator through a combination of molecular (broad-range 16S ribosomal RNA gene polymerase chain reaction - PCR) (2) and bacteriological methods (thioglycolate broth with resazurin and BHI broth to assess the growth of a wide range of aerobic and anaerobic bacteria) (3).

All experiments were performed in full compliance with the requirements of the French regional ethics committee number 19 (Comité d'Ethique en Expérimentation Animale Val de Loire) under the reference APAFIS#30655-2021042114338110 v1.

1.2. Tissue sampling

At day 21 post-hatching, conventional and germ-free birds (n=8 per group) were sacrificed by cervical dislocation followed by blood withdraw. Caecal contents and lungs were immediately collected from each chicken, snap frozen in liquid nitrogen and stored at -80°C for further metabolomics analysis and RNA extraction.

1.3. Metabolomics analysis

Caecal contents and lung samples were specifically prepared for SCFA quantification according to the protocol described by Hauser and colleagues with slight modifications (4). Briefly, caecal samples (20-50 mg) were homogenized in 1.2 ml of phosphate buffer (0.2 M, pH 7) and 1.2 ml of $CDCl_3/CD_3OD$ (2:1, v/v) solution. Samples were then centrifuged (5500 g, 10 min, 4°C) twice. The aqueous phases were collected, dried using a SpeedVac Vacuum Concentrator, reconstituted in phosphate buffer (0.2 M, pH 7), vortexed, and centrifuged (5500 g, 15 min, 4°C). Finally, 500 µl of supernatant were transferred into 5 mm NMR tubes and 100 µl of TSP (1 mM) were added to NMR tubes. Tissue samples (100-200 mg) were homogenized in 4,85 ml of methanol/water (80/20, v/v) per gram of tissue during 40 s. Next, 2 ml of dichloromethane per gram of tissue were added and the sample was vortexed (5 s). Finally, 2 ml of dichloromethane and 2 ml of water per gram of tissue were added and the sample was vortexed (10 s) and kept stand at 4°C during 15 min. The sample was then centrifuged (5500 g, 15 min, 4°C) and the aqueous phases were collected. Samples were dried using a SpeedVac Vacuum Concentrator, reconstituted in 200 µl of phosphate buffer (0.2 M, pH 7), vortexed and centrifuged (5500 g, 15 min, 4°C). Then, 150 µL of supernatant were transferred into 3 mm NMR tubes, and 50 µL of TSP solution (1 mM) were added to the NMR tube. ^{1}H NMR spectra

were obtained at 300 K on a Bruker Avance III HD 600 MHz NMR spectrometer (Bruker Biospin, Rheinstetten, Germany), operating at 600.13 MHz for ^{1}H resonance frequency using an inverse detection 5 mm ^{1}H-^{13}C-^{15}N-^{31}P cryoprobe attached to a Cryoplatform (the preamplifier unit). « Tuning » and « matching » of the probe, lock, shims tuning, pulse (90°) and gain computation were automatically performed for each sample. ^{1}H NMR spectra were acquired using the "noesypr1d" pulse sequence for water signal suppression, with a mixing time of 100 ms. A total of 1024 and 256 transients were collected for tissue and caecal samples, respectively, into 64k data points using a spectral width of 12 ppm, a relaxation delay of 15 s and an acquisition time of 4.5 s. Prior to Fourier transform, an exponential line broadening function of 0.3 Hz was applied to the FID. All NMR spectra were phase- and baseline-corrected and referenced to the chemical shift of TSP (0 ppm) using Topspin (V3.2, Bruker Biospin, Germany). Metabolites concentrations were calculated using the TSP signal at 0 ppm integrating for 9 protons and with a known concentration, and the signals corresponding to acetate (1.92; s), propionate (1.06; t) and butyrate (0.90; t).

1.4. Gene expression analysis

Total RNA was extracted from lung samples using the NucleoSpin RNA II kit (Macherey-Nagel, Germany) according to the manufacturer's instructions, including an rDNAse step for the elimination of contaminant DNA. RNA quality and concentration were determined using a NanoDrop (Thermo Scientific, USA). Total RNA (1 µg per reaction) was reverse transcribed using the iScript cDNA synthesis kit (Bio-Rad, USA). Quantitative real-time PCR (qRT-PCR) was performed on a CFX96 machine (Bio-Rad, USA), the reaction mixture consisting of iQ SYBR Green Supermix (Bio-Rad, USA), cDNA, primers (250 nM, Eurogentec, Belgium) and nuclease-free water (Sigma-Aldrich, UK) in a total reaction volume of 20 µL. qRT-PCR data were analyzed using the CFX Manager software 3.1 (Bio-Rad, USA). Amplicon size of qRT-PCR products was confirmed in a 2% agarose gel. Gene expression for each target gene was normalized to gene expression levels of chicken glyceraldehyde 3-phosphate dehydrogenase (GAPDH) and β-2-microglobulin (β2M). Relative normalized expression was calculated using the $2-\Delta\Delta Ct$ method and data are represented as Log2 fold change as compared to the conventional chicken group. Primer pairs used for the qRT-PCR analysis are show in the **Supplementary Table 1.**

Statistical analysis

Data are expressed as the median. Statistical analysis was performed by a nonparametric Mann-Whitney U test (between two groups) or one way ANOVA with post hoc Tukey test (between more than 2 groups), using Graph Pad Prism 8.0 software (GraphPad, San Diago, CA, USA). Statistical significance was set at $p < 0.05$.

Supplementary Table 1. Primer pairs utilized for the qRT-PCR analysis in the present study.

Target genes	Forward primers 5'-3'	Reverse primers 5'-3'	Amplicon size (bp)
AP1	TCCCCTGTCCCCTATTGACA	CGCCGCAATTCTGTTTCTCA	79
B2M	CGTCCTCAACTGCTTCGCG	TCTCGTGCTCCACCTTGC	194
CCR2	ATGCCAACAACAACGTTTGA	TGTTGCCTATGAAGCCAAA	127
CD14	CATGCTTGGCAGTCTGCAAA	CAGGAGGACCTCAGGAACCA	67
CSF1	GCGACTCTGTCTGCTACGTG	CGAAGGTCTCCTTGTTCTGC	291
FLT3	CATTCGGACCCAGTACATGTTTAC	TGAGCCGTAGAAGAGCAGGTATAA	78
GAPDH	GTCCTCTCTGGCAAAGTCCAAG	CCACAACATACTCAGCACCTGC	223
IKKA	CTTTCATCTATGGCAACTCCTG	ATGTCCAAACCAAGACGTGAT	244
IKKB	TGACGCTGTAGTTGCAGACACA	TCCGCAGCTGGAAAAAGTG	152
IL10	CACAACTTCTTCACCTGCGAG	CATGGCTTTGTAGATCCCGTTC	96
MAFB	AGGACCGGTTCTCGGATGAC	CCTCGGAGGTGCCTGTTG	67
MRC1LB	GGAACTGCAGGCAGCATATGT	CACAAGGTGCACCAAAATTATTTC	79
MYD88	AGAAGGTGTCGGAGGATGGTG	GGGCTCCAAATGCTGACTGC	365
NOS2	CCACCAGGAGATGTTGAACTATGTC	CCAGATGTGTGTTTTCCATGCA	76
STAT1	AAGCAAACGTAATCTTCAGGATAAC	TTTCTCTCCTCTTTCAGACAGTTG	87
TLR15	AGCTGAACTGCTGCCACATTT	TTTCCTCTGTTCTTCTTTGTCTGAATC	83
TLR3	AACACCCCGCCTAAATATCA	CCACCCTTCAAAATGGATGA	153
TLR4	ATCTTTCAAGGTGCCACATC	GGATATGCTTGTTTCCACCA	167

Supplementary References:

1. Guitton E, Faurie A, Lavillatte S, Chaumeil T, Gaboriaud P, Bussière F, Laurent F, Lacroix-Lamandé S, Guabiraba R, Schouler C. Production of Germ-Free Fast-Growing Broilers from a Commercial Line for Microbiota Studies. *JoVE* (2020)61148. doi: 10.3791/61148

2. Zhu XY, Zhong T, Pandya Y, Joerger RD. 16S rRNA-based analysis of microbiota from the cecum of broiler chickens. *Appl Environ Microbiol* (2002) **68**:124–137. doi: 10.1128/AEM.68.1.124-137.2002

3. Brewer JH. CLEAR LIQUID MEDIUMS FOR THE "AEROBIC" CULTIVATION OF ANAEROBES. *JAMA* (1940) **115**:598. doi: 10.1001/jama.1940.72810340001009

4. Hauser A, Eisenmann P, Muhle-Goll C, Luy B, Dötsch A, Graf D, Tzvetkova P. Efficient Extraction from Mice Feces for NMR Metabolomics Measurements with Special Emphasis on SCFAs. *Metabolites* (2019) **9**:E55. doi: 10.3390/metabo9030055
